# Characterizing Complications of Deep Brain Stimulation Devices for the Treatment of Parkinsonian Symptoms Without Tremor: A Federal MAUDE Database Analysis

**DOI:** 10.7759/cureus.15539

**Published:** 2021-06-09

**Authors:** Josiah Bennett, Jack MacGuire, Ena Novakovic, Huey Huynh, Keri Jones, Julian L Gendreau, Antonios Mammis, Mickey E Abraham

**Affiliations:** 1 Neurological Surgery, Mercer University School of Medicine, Savannah, USA; 2 Neurological Surgery, Mercer University School of Medicine, Macon, USA; 3 Graduate Medical Education, Eisenhower Army Medical Center, Augusta, USA; 4 Biomedical Engineering, Johns Hopkins University, Baltimore, USA; 5 Neurological Surgery, New York University School of Medicine, New York, USA; 6 Neurological Surgery, University of California San Diego, San Diego, USA

**Keywords:** adverse events, complications, deep brain stimulation, fda, maude, parkinson’s disease

## Abstract

Introduction

Deep brain stimulation (DBS) is a modality of treatment for medication refractory Parkinson’s disease (PD) in patients with debilitating motor symptoms. While potentially life-changing for individuals with Parkinson’s disease, characterization of adverse events for these DBS devices have not yet been systematically organized. Therefore, the goal of this study was to characterize reported complications of DBS devices reported to the Food & Drug Administration over the last 10 years.

Methods

The Manufacturer and User Facility Device Experience (MAUDE) database was utilized to retrieve entries reported under “Stimulator, Electrical, Implanted, For Parkinsonian Symptoms” between July 31, 2010 and August 1, 2020. After removing duplicate entries, each unique adverse event reported was sorted into complication categories based on the entries’ provided narrative description. A final tabulation of complications was generated.

Results

The search query revealed 221 unique adverse events. The most common DBS devices were the Vercise Gevia, Vercise Cartesia and Vercise PC produced by Boston Scientific (Marlborough, MA, USA). The most commonly reported complications were infection (16.2%) follow by lead migrations (8.6%). Other common causes of complications were circuit-related impedance (6.5%), cerebral bleeds (6.3%), device failure (6.3%) and device-related trauma (4.5%). Over a third (40%) of all devices reported with adverse events required returning to the operating room for explant or revision.

Conclusion

The most common complications of DBS systems are infections followed by lead migrations. Further research is needed to minimize infection rates associated with DBS systems and to reduce intrinsic device malfunctions for patients in the future.

## Introduction

Parkinson’s disease (PD) is the fastest-growing cause of neurologic related disability with a prevalence exceeding six million individuals [1]. It is a progressive disease eventually leading to significant disability and handicap for patient due to the loss of motor function and neurocognitive sequelae [2,3]. Initial steps in treatment include both non-pharmacologic and pharmacologic approaches, which can include physical therapy and administration of dopamine receptor agonists. Unfortunately, a small number of patients eventually sustain progressive disease that fails medical management and acquires debilitating symptomatology that leads to a significant reduction in quality of life. These patients are potential candidates for deep brain stimulation (DBS), which has been shown to improve motor qualities that have been affected by the progression of PD more than pharmacologic therapy alone in randomized control trials [4-7]. Symptomology of PD that is not affected by dopaminergic agonists, such as PD-associated dementia, are also not affected by DBS [8-10].

DBS utilizes electrical stimulation of either the subthalamic nucleus (STN) or globus pallidus internus (GPi) requiring unilateral or bilateral electrode placement. DBS is reversible but does produce permanent brain tissue injury, which is a large reason as to why it has mostly replaced permanent lesioning as a treatment for PD [7]. DBS was also found to offer increased clinically significant motor improvement when compared to individuals treated with medical therapy alone [3]. Therefore, DBS has the potential of significantly improving the quality of life for patients who are candidates to undergo a surgical operation [11].

As this is a promising modality of future control in PD, surveillance and safety considerations for this device should be paramount. Especially as there is an ongoing effort to improve vigilance of medical devices after initial FDA approval and that complications of DBS have been shown to have almost double the cost than uncomplicated cases of DBS [12,13]. This data is already reported for other neuromodulation devices such as occipital nerve stimulators, responsive neurostimulation and dorsal root ganglion stimulators [14-16]. Therefore, the purpose of this study was to categorize reported complications of DBS devices submitted to the United States Food & Drug Administration (FDA). To accomplish this goal, the federal Manufacturer and User Facility Device Experience (MAUDE) database was queried for all entries reporting complications of the DBS system for PD during this timeframe [17].

## Materials and methods

The Manufacturer and User Facility Device Experience database

The MAUDE database is maintained by the United States FDA to allow for public viewing of all reported complications of medical devices approved in the United States. It is maintained by the FDA for the purpose of post-hoc review of device safety [17]. There are both mandatory reporters and voluntary reporters in the database. Healthcare facilities using these devices and the manufacturers of medical devices are required to report adverse events of their medical devices for public viewing. Manufacturers often rely on reports from the treating institutions and medical providers to report complications, thus not all complications are adequately reported to this database through mandatory reporting. Additionally, patients, families and individual healthcare providers also have the option to submit reports on a voluntary basis. With these limitations of underreporting adverse events, the FDA recommends against using the database for the purpose of generating accurate numbers of incidence and prevalence. However, the database can be an effective tool for characterizing the unique complication types and also comparing rates of complications relatively among other complications associated with the device.

Data search protocol

The MAUDE database was queried for all entries listed under “Stimulator, Electrical, Implanted, For Parkinsonian Symptoms,” during the time period of July 31, 2010-August 1, 2020. The last ten years were queried only, as we wanted to obtain documented complications related to the most updated DBS devices. The authors felt that searching from its initial approval in 2002 would reveal many complications that would occur due to outdated equipment.

It is important to note that there is a separate category of “Stimulator, Electrical, Implanted, For Parkinsonian Tremor” that was not queried in this study that consisted of manufacturers such as Abbot (Chicago, Illinois, USA), St Jude Medical (Saint Paul, Minnesota, USA) and Medtronic (Dublin, Ireland). Therefore, this study only analyzed complications of DBS implantations for primarily non-tremor Parkinsonian symptoms only.

Entries with identical event report numbers and identical event descriptions were considered duplicate entries and removed. Each component of the device was often reported differently as separate entries (a single adverse event involving one lead and one generator event could be reported as two separate reports). These were identified by a linking reference number provided in the event description.

Classification of complications

Categories of complications were devised by the authors JB and JM (Table 1). From these categories, complications were also stratified into subtypes. All complications that resulted in explantation, revision, or other management were recorded.

## Results

The Manufacturer and User Facility Device Experience database search

A total of 218 entries were reported to the MAUDE database with complication dates occurring between July 31, 2010 and August 1, 2020. The entries reported by the MAUDE database showed four duplicates that were removed. These were removed, yielding a total of 214 entries.

**Figure 1:**
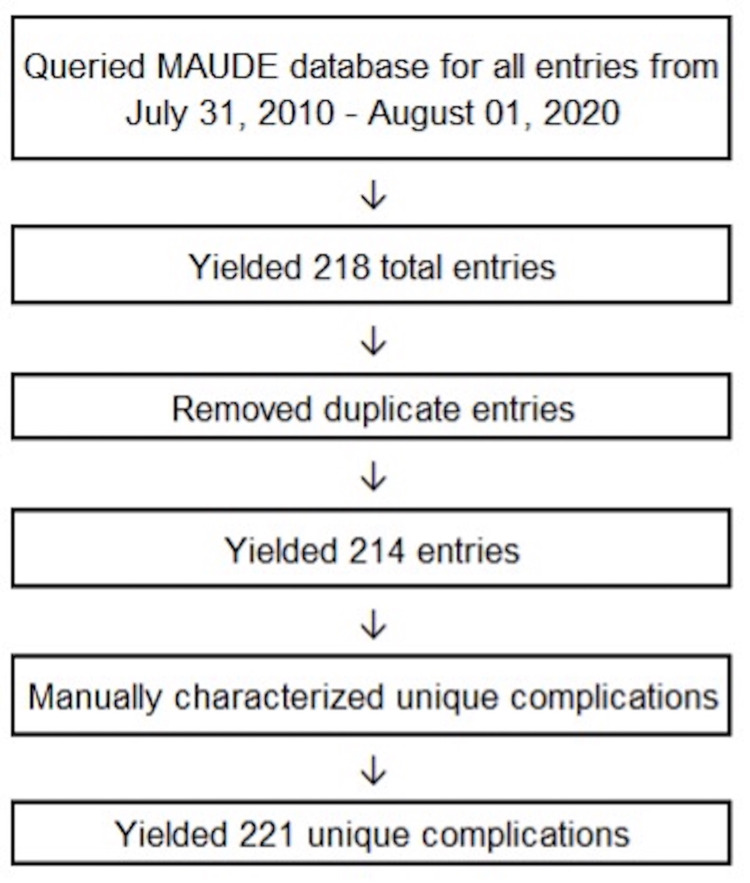
Flowchart of the data selection process, the elimination of duplicate entries, and the characterization of unique complications.

Manual characterization of complications revealed cases where numerous complications were assigned to the same patient and reported together on a single date. When multiple adverse occurred for the same patient secondary (e.g., a patient experienced a lead fracture and also infection), they were each categorized as unique complications. Seven entries were found to have contained two unique complications. After correction for additional complications, 221 total unique complications remained for analysis. A flowsheet of the data extraction and selection is displayed in Figure 1. Categorization of the remaining filtered complications are characterized in Table 1. The most common brand names were Vercise Gevia, Vercise Cartesia and Vercise PC (Boston Scientific, Marlborough, MA, USA).

**Table 1:** Number of entries for each complication category. CSF: cerebrospinal fluid.

Characterization	Complications	Number of complications
Procedural (101)	Infections	36
	Cerebral bleeds	14
	Lead placement	11
	Cerebral edema	10
	Wound dehiscence	8
	Seizure	5
	CSF leak	3
	Pneumocephalus	2
	Inadequate stimulation	2
	Other trauma	10
Device-related malfunction (49)	Lead fracture	5
	Intrinsic device failure	14
	Migration	19
	Impedance	11
Stimulation-related complications (31)	Inadequate stimulation and pain relief	10
	Worsening Parkinson symptoms	8
	Seizure	6
	Focal neurological side effects	5
	Burning at incision site	1
	Lumbosciatica	1
Other patient complaints (13)	Neurological symptoms	8
	Pain at site	5
Other (27)	Multifarious	21
	External trauma	6

Surgical complications

**Figure 2:**
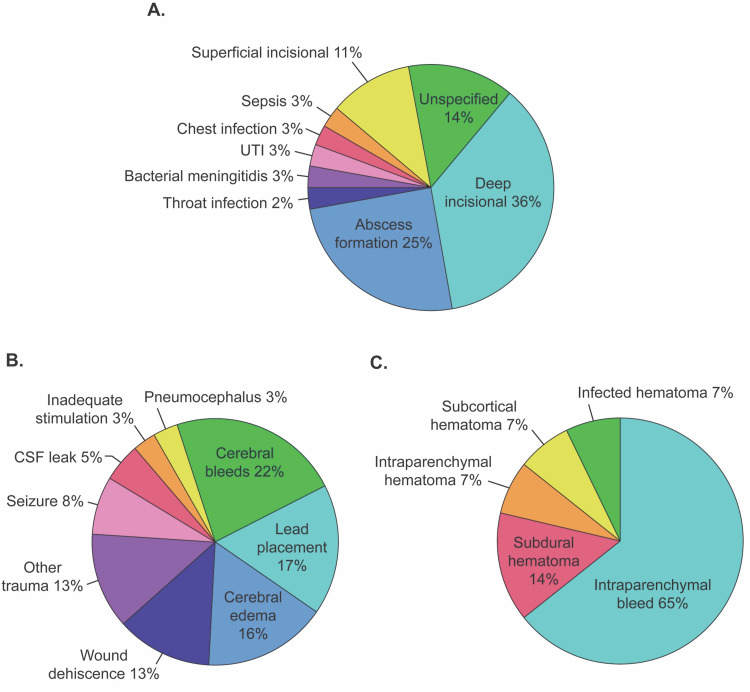
Stratification of infectious procedural complications, noninfectious procedural complications and cerebral bleeds. (A) Breakdown of infectious procedural complications. Entries shown as superficial and deep incisional were described as such in their reports, without indicating specific infection types. (B) Breakdown of non-infectious procedural complications. Cerebral bleeds accounted for 22% of noninfectious procedural complications. (C) Breakdown of cerebral bleeds. Intraparenchymal accounted for more than half of cerebral bleeds. UTI: urinary tract infection; CSF: cerebrospinal fluid.

The most common surgical complication was infection (36.4%). The specific detail and location of infection varied among reports. Some entries described the exact location of infection (e.g., IPG site) whereas others were generalized to area (e.g., chest). After tabulating infection type by location, deep incisional at IPG pocket site was reported at the highest rate (36.1%), followed by intracerebral abscess (25.0%). There were four complications of superficial infection at the incision site of skull (11.1%). The remaining infections were unspecified (13.9%) or due to other unique causes such as throat infection (2.8%), bacterial meningitis (2.8%), urinary tract infection (2.87%), chest infection (2.8%), and sepsis (2.8%). Unspecified entries were categorized as such due to lack of specific location, appropriate treatment, or infection source (eg, lack of positive or negative blood culture for bacteria) (Figure 2A). For management of the infectious complications, the majority resulted in explant (63.9%) and other treatments such as antibiotics and steroids (27.8%). Four infections were managed with a revision (11.1%).

 

Noninfectious causes comprised 63.6% of the complications related to the original implant surgery. Cerebral bleeds accounted for 22% of all noninfectious complications, followed by lead placement (17%), cerebral edema (16%), wound dehiscence (12.7%), seizure (7.9%), CSF leak (4.8%), pneumocephalus and inadequate stimulation (3.2% each), and other trauma (12.7%; Figure 2B). Intraparenchymal bleed accounted for 64.3% of cerebral bleeds, subdural hematoma accounted for 14.3%, and intraparenchymal, subcortical, and infected hematoma made up 21.4% (Figure 2C). 

There were no instances of death reported by the MAUDE database that could be associated with the DBS system during the implant procedure.

Device-related complications

The most reported device-related complications were migration of device leads, accounting for 38.8%. Complications due to migration resulted in 11% system explants, 47% revisions, and 42% managements with other means (e.g., replacement). The second most common device-related complications were those related to device failure such as refractory battery failure, signal activation failure, inadequate stimulation, etc. (28.6% of all device-related complications). Impedance (attenuated electrical current) and lead fracture accounted for the remaining device-related complications with 22.4% and 10.2%, respectively.

Neurostimulation-related symptoms

Neurostimulation-related symptoms included inadequate stimulation and pain relief, worsening PD symptoms, seizure, focal neurological side effects, burning at incision site, and lumbosciatica. Of the 31 neurostimulation-related symptoms, inadequate stimulation and pain relief were the number one complaint, with 32.3%. This resulted in 40% explant of system device, 20% revision, and 40% other managements. Worsening PD symptoms accounted for 25.8%, with no reported explants or revisions. Seizure attributed 19.4% to the total device-related symptoms, and focal neurologic side effects stood at 16.1%. Burning at incision site and lumbosciatica made up 3.2% each, none with explants.

Patients complaints

There were 13 patient complaints that occurred at a length of time after implantation, with eight complaints of neurological symptoms and five complaints of pain. Neurological symptoms included anxiety (1), headache (1), panic attack (1), pain at site (1), stroke (1), back pain (1), gait disorder and episodes of "freezing" (1), delirium (1), impulsivity (1), while pain included neck discomfort (1), pain while attempting to stand up from sitting position (1), chest pain (1), discomfort at clip anchor site (1), and pain at IPG site (1). The one case of delirium spontaneously resolved. The patient who experienced refractory headaches despite reprogramming had his device explanted. Symptoms of pain improved with revision of device and/or pain medication.

Other complications

There were 27 other complications that were not categorized by the categories above, with 78% unspecified complications and 22% external head traumas. External head traumas included fall (3), dog-related injury (1), haircut (1), and head banging (1). The patient whose complication resulted from head banging received IV antibiotics for six weeks and underwent explant procedure during which a cranial lead and lead extension were removed and the infection resolved.

Surgically managed complications

Of the 221 unique complications, 60% were treated by other managements, while 24% resulted in explant and 16% in revision. Infectious procedural complications required the greatest number of explants (43%), migration held the number one spot for revision (25%), and among treatments with other managements, the majority was cerebral bleeds (9%). Most of the infectious procedural complications required either device explant or revision (75%), with device explant contributing 64%. In contrast, only 17% of noninfectious procedural complications (n = 65) resulted in either device explant or revision, and the remaining 83% were managed by other means such as antibiotics and steroids. This is depicted in Table 2.

**Table 2:** Treatments listed in the event narratives for each entry. CSF: cerebrospinal fluid.

Characterization	Complications	Explant	Revision	Other managements
Procedural	Infections	23	4	9
	Cerebral bleeds	0	2	12
	Lead placement	0	3	8
	Cerebral edema	0	0	10
	Wound dehiscence	1	2	5
	Seizure	0	0	5
	CSF leak	0	0	3
	Pneumocephalus	0	0	2
	Inadequate stimulation	1	1	0
	Other trauma	0	1	9
Device-related malfunction	Lead fracture	1	3	1
	Device failure	3	5	6
	Migration	2	9	8
	Impedance	2	3	6
Stimulation-related complications	Inadequate stimulation and pain relief	4	2	4
	Worsening Parkinson symptoms	0	0	8
	Seizure	0	0	6
	Focal neurologic side effects	0	0	5
	Burning at incision site	0	0	1
	Lumbosciatica	0	0	1
Other patient complaints	Neurological symptoms	1	0	7
	Pain at site	0	0	5
Other	Multifarious	11	1	9
	External trauma	4	0	2
Total		53	36	132

## Discussion

Overall, the most common procedural-related complications were infections. During review of the adverse event narrative reports, neurosurgeons attributed the majority of infections to the original implantation surgery. Many routes can be used to achieve lower infection rates in this population of patients. Optimal adherence to the sterile procedure has been associated with improved infection outcomes [18]. For DBS, Kozano et al hypothesize that a shorter duration between surgeries in two-stage surgeries for electrode placement can lead to increased likelihood of infection [19]. Kim et al found that infection was associated with intensive care unit stay after surgery and also with shorter antibiotic prophylaxis after surgery [20]. Smoking was found to increase the likelihood of infection and patients should be counseled for cessation prior to undergoing surgery [21]. Finally, it has been found that surgical site infections can occur even after 90 days postoperatively, therefore DBS implantation patients should ultimately be followed for at least one year postoperatively [22].

Lead migrations comprised the second most common complication patients experienced with DBS. In a retrospective study of 138 leads in 132 patients with both initial and delayed localization CT scans by Morishita et al, over 10% of DBS leads were displaced by greater than 3 mm on delayed measurement. This is comparable as the rate of lead migrations found in our study of 8.6% with the slightly lower reported number in our study be likely due to lead migration events going unreported if not clinically significant. These were found to be associated with technical error during implantation of the DBS pulse generator and failure of fixation at the burr hole [23]. White-Dzuro et al proposed a novel technique utilizing a titanium hemoclip and cement to secure leads which reduced the number of complications in their series of 291 patients [24]. Additionally, microtextured features on the surface of the DBS probe can potentially be used to minimize the extent of electrode migration [25].

The next most reported complications were bleeds. Most hemorrhages were either at the skin or located subcortically; with reports of intracerebral hemorrhages and hemorrhages located in the subthalamic nucleus ranging from 2% to 7.2% in previous clinical reports [26,27]. Hemorrhages have been found to occur more often in patients with hypertension, suggesting optimal blood pressure control will minimize intra-operative and post-operative hemorrhages [28]. The Neurostimulation Appropriateness Consensus Committee (NACC) recommends discontinuing aspirin six days prior to surgery and discontinuing non-selective nonsteroidal anti-inflammatory drugs five half-lives ahead of surgery. Selective Cox-2 inhibitors do not need to be discontinued prior to surgery per these guidelines [29].

After bleeds, follow both intrinsic device failure and device impedance. In study period spanning a total of four years, DBS for PD was found to be discontinued in 6.1% of patients secondary to intrinsic device malfunction [28]. When reviewing narrative reports provided by the MAUDE database it was reported that high impedance found in leads postoperatively were an indicator of device malfunction or an infectious process. Since the MAUDE database is self-reported, it is inappropriate to discern precise data, but increases in impedance should be closely monitored as an early indication of device malfunction or the development of an abscess formation or other infectious etiology. However, impedance itself should decrease over time while still providing benefit for the patient in terms of motor function even after 13 years [30].

Finally, neurological deficits and patient complaints were also commonly reported complications postoperatively. There were no complications of depression, suicide or confusion reported to the MAUDE database; however, these conditions are prevalent in patients with PD (depression: 1%-12%, suicide: 0%-1%, confusion: 1%-13%) [5, 31-34]. This rate of psychiatric conditions is likely not to be attributed to the DBS system specifically, but rather than comorbidities of PD itself. Additionally, PD patients are found to have poorer neuropsychological testing not reported by this database [35]. Interestingly, DBS is associated with clinically debilitating dyskinesia, which was not reported by the database [36].

When compared with other neuromodulation devices, it appears that infections are generally the same rate as occipital nerve stimulators and dorsal root ganglion stimulators and less than responsive neurostimulation (16.2% vs 40%). However, responsive neurostimulation has less lead migrations (8.6% vs 2%). Compared to occipital nerve stimulators and dorsal root ganglion stimulators (8.6% vs 17 - 28%) deep brain stimulation has less migrations. This is likely due to the depth electrodes required for DBS deep into the brain parenchyma and due to the more rigid fixation of the depth electrode to the skull.

Limitations

The MAUDE database is a self-reporting database; thus, health care providers are not mandated to report adverse events to it. Therefore, this database underestimates incidence rates of specific adverse events as well as not representing accurate data for rates of specific adverse events. Subjective complaints including quality of life issues with the device may be even more underreported. This is why the FDA does not recommend calculating incidence rates from data from the MAUDE database.

Furthermore, duplicate reports may have been missed due to sources reporting the same complications with different descriptions. Despite taking care to remove duplicates of event reports to the MAUDE database, it is possible duplicates still remain.

Comorbidities were not reported to the MAUDE database. Therefore, this factor could not be controlled for when looking at complications. For example, patients who experienced hemorrhages could have had a comorbid bleeding disorder or a hypercoagulable state which would have increased the likelihood of the hemorrhage or other hematologic complication.

Complications for DBS systems used primarily to treat Parkinsonian tremor specifically were listed in a separate subset in the MAUDE dataset. Therefore, this study largely only describes DBS devices produced by Boston Scientific as other manufacturer’s DBS devices were listed in this separate subset. Future work should be performed assessing complications of the DBS device for all indications of DBS.

Finally, the MAUDE database is only reporting adverse events and does not measure how effective DBS was for improving the motor symptoms of PD, so making conclusions on clinical outcomes is inappropriate from the MAUDE database.

## Conclusions

For DBS, the most commonly reported complications are procedural-related infections, which were most often treated with device explant. Lead migration was the second most common complication of the DBS system. Future investigations of improvement for the device should aim to reduce infections and lead migrations by improving the implantation procedure or by altering the physical properties of device during production.
