# Correction: Characterizing Complications of Deep Brain Stimulation Devices for the Treatment of Parkinsonian Symptoms Without Tremor: A Federal MAUDE Database Analysis

**DOI:** 10.7759/cureus.c46

**Published:** 2021-08-17

**Authors:** Josiah Bennett, Jack MacGuire, Ena Novakovic, Huey Huynh, Keri Jones, Julian L Gendreau, Antonios Mammis, Mickey E Abraham

**Affiliations:** 1 Neurological Surgery, Mercer University School of Medicine, Savannah, USA; 2 Neurological Surgery, Mercer University School of Medicine, Macon, USA; 3 Graduate Medical Education, Eisenhower Army Medical Center, Augusta, USA; 4 Biomedical Engineering, Johns Hopkins University, Baltimore, USA; 5 Neurological Surgery, New York University School of Medicine, New York, USA; 6 Neurological Surgery, University of California San Diego, San Diego, USA

Unfortunately, after publication of this article, the authors noticed that Brian Walker's name was associated with Boston Scientific in the abstract. It was determined his name was written in a way that suggested he was involved in the production of these devices. However, he is only a sales representative for the organization. Therefore, it was removed from the article post-publication. The authors apologize for this oversight.

